# Disease and the Weather

**Published:** 1872-11

**Authors:** 


### Disease and the Weather.

A writer in the *British Medical Journal* remarks:

"Meteorologists all knew that the warm and most equatorial current is generally accompanied in winter by a decrease of barometric pressure. How cleverly did the great Jenner embody in a few lines of verse, "On the Signs of Rain," the effects of these atmospheric changes:

"Hark! how the chairs and table crack,
Old Betty's joints are on the rack."

The aching of corns is as readily accounted for as the cracking of chairs and tables; for both are evidentally the result of the increase of the amount of moisture in the air. The great alteration in size which epithelial and ligneous structures undergo by the addition or subtraction of moisture are well known; what are the changes in the body when it is exposed to low barometric pressure? Richardson, Hewson, and others tell us that there is a tendency to exudation of fluid from wounded surfaces, a feebleness in the healing of wounds, a susceptibility to disturbance in the body generally, and a proneness to the production of secondary fever by the absorption of discharges which have undergone some decomposition. The outcome of these facts has been the establishment of the law, that no important surgical operation should be performed when the barometer is low, or when it is steadily falling. The principal effect, however, of diminished pressure of the atmosphere is distension of the capillaries. We all recognize as one of the exciting causes of apoplectic seizures a rapid diminution of atmospheric pressure, producing a sudden capillary engorgement. An exacerbation of the symptoms in cases of joint-disease may be due, perhaps, to the same meteorological change acting in a manner which may be thus explained. In the solid inelastic articular expansions of the bones, which are surrounded by firm inextensible textures forming the joints, the minute nerves, shown by Kolliker and others to permeate the cancellous and compact structures in company with vessels, are pressed by these vessels when enlarged against the unyielding walls of the channels through which they pass. Although the nerves of bones do not generally afford healthy individuals any conscious sensations, yet, in diseases of the joints, the bones, when congested or the seat of inflammation, become painful. Tissues not supplied with rigid canals like bone, yield to pressure during any temporary increase in the size of the minute vessels. In such tissues, vascular distension from a diminution of the pressure of the air is unaccompanied by pain, because the nerves accompanying the vessels are uninterfered with. Low barometric pressure and an excess of humidity of the air offer conditions most favorable for the removal of heat by evaporation and radiation from a congested or an inflamed joint. Teeth, which have a nutrient system very similar to that possessed by bone, become painful when the pressure of the air is suddenly lessened for the same reason. The nerves of the tooth being in a morbid condition from caries, are temporarily irritated by the capillary enlargement."

